# Risk of major cardiovascular events in patients with psoriasis receiving biologic therapies: a prospective cohort study

**DOI:** 10.1111/jdv.16018

**Published:** 2019-11-19

**Authors:** W. Rungapiromnan, K.J. Mason, M. Lunt, K. McElhone, A.D. Burden, M.K. Rutter, R.B. Warren, C.E.M. Griffiths, D.M. Ashcroft, BADBIR Study Group, Jonathan Barker, Marilyn Benham, Fiona Browne, Ian Evans, Sagair Hussain, Brian Kirby, Linda Lawson, Tess McPherson, Ruth Murphy, Caroline Owen, Anthony Ormerod, Eleanor Pearson, Nick Reynolds, Josh Richards, Catherine Smith

**Affiliations:** ^1^ Centre for Pharmacoepidemiology and Drug Safety School of Health Sciences Faculty of Biology, Medicine and Health University of Manchester Manchester UK; ^2^ Division of Musculoskeletal and Dermatological Sciences School of Biological Sciences Faculty of Biology, Medicine and Health University of Manchester Manchester UK; ^3^ Institute of Infection, Immunity and Inflammation University of Glasgow Glasgow UK; ^4^ Division of Endocrinology, Diabetes and Gastroenterology School of Medical Sciences Faculty of Biology, Medicine and Health University of Manchester Manchester UK; ^5^ Manchester Diabetes Centre Manchester University NHS Foundation Trust Manchester Academic Health Science Centre Manchester UK; ^6^ Dermatology Centre Salford Royal NHS Foundation Trust University of Manchester Manchester Academic Health Science Centre Manchester UK

## Abstract

**Background:**

The cardiovascular safety profile of biologic therapies used for psoriasis is unclear.

**Objectives:**

To compare the risk of major cardiovascular events (CVEs; acute coronary syndrome, unstable angina, myocardial infarction and stroke) in patients with chronic plaque psoriasis treated with adalimumab, etanercept or ustekinumab in a large prospective cohort.

**Methods:**

Prospective cohort study examining the comparative risk of major CVEs was conducted using the British Association of Dermatologists Biologics and Immunomodulators Register. The main analysis compared adults with chronic plaque psoriasis receiving ustekinumab with tumour necrosis‐α inhibitors (TNFi: etanercept and adalimumab), whilst the secondary analyses compared ustekinumab, etanercept or methotrexate against adalimumab. Hazard ratios (HRs) with 95% confidence intervals (CIs) were calculated using overlap weights by propensity score to balance baseline covariates among comparison groups.

**Results:**

We included 5468 biologic‐naïve patients subsequently exposed (951 ustekinumab; 1313 etanercept; and 3204 adalimumab) in the main analysis. The secondary analyses also included 2189 patients receiving methotrexate. The median (p25–p75) follow‐up times for patients using ustekinumab, TNFi, adalimumab, etanercept and methotrexate were as follows: 2.01 (1.16–3.21), 1.93 (1.05–3.34), 1.94 (1.09–3.32), 1.92 (0.93–3.45) and 1.43 (0.84–2.53) years, respectively. Ustekinumab, TNFi, adalimumab, etanercept and methotrexate groups had 7, 29, 23, 6 and 9 patients experiencing major CVEs, respectively. No differences in the risk of major CVEs were observed between biologic therapies [adjusted HR for ustekinumab vs. TNFi: 0.96 (95% CI 0.41–2.22); ustekinumab vs. adalimumab: 0.81 (0.30–2.17); etanercept vs. adalimumab: 0.81 (0.28–2.30)] and methotrexate against adalimumab [1.05 (0.34–3.28)].

**Conclusions:**

In this large prospective cohort study, we found no significant differences in the risk of major CVEs between three different biologic therapies and methotrexate. Additional studies, with longer term follow‐up, are needed to investigate the potential effects of biologic therapies on incidence of major CVEs.

## Abstract 2

**Linked Commentary:** K. Kridin and A.D. Cohen. J Eur Acad Dermatol Venereol 2020; 34: 668–669. https://doi.org/10.1111/jdv.16345.

## Introduction

Psoriasis is a common, chronic inflammatory skin disease affecting over 125 million people worldwide.^1^ The prevalence of psoriasis varies between countries (0.91–8.5%), and recent estimates suggest that almost 3% of the UK population are affected by the disease.^2^, ^3^ Cardiovascular (CV) comorbidities are common among patients with psoriasis.^4^ Moreover, CV risk factor screening of adult patients with psoriasis in primary care has found a high proportion of patients being sub‐optimally treated for known CV risk factors.^5^ This can contribute to an increased risk of major CV events (CVEs) in patients with psoriasis.

Biologic therapies are increasingly used for the treatment of moderate–severe psoriasis, but their CV safety profile is still unclear. In recent years, concerns have been raised regarding an increased CV risk due to the use of anti‐interleukin (IL)‐12/23 agents after a number of major adverse CVEs s [MACEs; myocardial infarction (MI), cerebrovascular accident or CV death] occurred in patients receiving briakinumab [anti‐IL‐12/23 agent; Five patients experiencing major adverse CVEs (onset ranged from 21–55 days) during the induction phase and two patients experiencing the events on day 131 and 225 during the maintenance phase] which in part resulted in the discontinuation of the development of this treatment.^6^, ^7^, ^8^ A recent meta‐analysis of randomized controlled trials (RCTs) suggested that there was no significant difference in the risk of MACEs between licensed biologic therapies and placebo.^9^ However, the risks were examined over short periods (10–30 weeks) and participants included in RCTs tend to have fewer comorbidities than psoriasis patients in a real‐world setting.^9^, ^10^ Several cohort studies have examined the impact of biologic therapies on CVEs in patients with psoriasis involving a range of different reference treatments including non‐biologic, non‐systemic therapies (topical therapy, phototherapy and climate therapy) or methotrexate.^11^, ^12^, ^13^, ^14^, ^15^ These therapies are typically recommended for patients before receiving biologic therapies. To assess the association between CVEs and treatments, participants in treatment and reference groups should have a similar severity of psoriasis since this may influence the development of CVEs.^16^ Ideally, biologic therapies should be directly compared.

The objectives of this study were to directly compare the risk of major CVEs (acute coronary syndrome, unstable angina, MI and stroke) in adult patients with chronic plaque psoriasis under routine care treated with adalimumab, etanercept or ustekinumab in a large prospective cohort using the British Association of Dermatologists Biologics and Immunomodulators Register (BADBIR).

## Methods

The BADBIR is a large prospective cohort study examining the long‐term safety of biologic therapies in patients with psoriasis. It compares a cohort of psoriasis patients treated with biologic therapies and a cohort of those treated with conventional systemic therapies (e.g. methotrexate). Data have been collected on patients with moderate–severe psoriasis being treated at 160 secondary care dermatology centres across the UK and the Republic of Ireland since September 2007. BADBIR was approved by the NHS Research Ethics Committee North West England (reference 07/MRE08/9) in March 2007, and all patients have provided written informed consent for participation. Further details regarding study design of BADBIR has been published previously.^17^

### Baseline assessments

Baseline data collected at enrolment include patient demographic characteristics, comorbidities, anthropometric data, drug therapies and clinical data such as type and severity of psoriasis (Psoriasis Area and Severity Index; PASI) by healthcare professionals using an online database, whilst lifestyle information such as smoking and alcohol consumption was collected directly from patients using a questionnaire.

### Follow‐up assessments

Data are collected every 6 months for the first 3 years and then annually. These include information on changes in drug therapies, measures of disease severity, hospitalization and details of adverse events (AEs) including the outcomes of interest of this study. Patient death details are derived from the BADBIR register via linkage with the Office of National Statistics mortality records. AEs are coded using the Medical Dictionary for Regulatory Activities (MedDRA) system.^18^

### Study population and exposure

Patients who enrolled in the BADBIR from September 2007 to October 2016 and had at least 6 months of follow‐up data following initiation of treatment were selected for this study. Biologic‐naïve patients aged at least 18 years old with chronic plaque psoriasis who had no prior history of major CVEs were selected for the inclusion in this cohort study. For the main analysis, patients receiving the first‐line originator anti‐IL‐12/23 agent (ustekinumab) were compared with TNFi (etanercept or adalimumab) as the reference group. For the secondary analyses, patients receiving first‐line adalimumab (the referent group) were compared with ustekinumab, etanercept or methotrexate.

### Outcome of interest and ascertainment

The outcome of interest was fatal or non‐fatal major CVEs [acute coronary syndrome, unstable angina, MI or stroke (Table S1, Supporting Information) provides the relevant MedDRA outcome codes]. All relevant MedDRA codes or descriptions of events were identified by WR. Both codes and descriptions were independently reviewed by a clinician with extensive experience in managing CV disease (MKR) in order to ascertain the final outcome of the study. To validate all serious outcomes, the BADBIR staff members asked study sites to confirm these events. Moreover, patients experiencing acute coronary syndrome, unstable angina and MI were also collected information on cardio marker, electrocardiogram, previous history of CV diseases, the use of thrombolysis and angioplasty and cardiac intervention, whilst patients experiencing stroke were also collected information on type of stroke, computed tomography scan and history of thrombolysis and atrial fibrillation in order to confirm these events.

### Data analysis

Patients were observed from the date of receiving therapy to developing the first major CVE or the earliest date of change in treatment (changing to other biologic therapy in the biologic cohorts or starting a biologic therapy in the methotrexate cohort); end of recorded data in the BADBIR; death; or end of the study follow‐up (30 September 2016). Discontinuation of treatment was defined as a gap in a regimen for more than 90 days. We examined the risk of major CVEs occurring over two periods: (i) whilst exposed to treatment; and (ii) extending the exposure effect window until 90 days after the last dose. Planned secondary analyses included direct comparisons between the individual biologic therapies and users of methotrexate.

Descriptive statistics were used to analyse baseline patient characteristics. Frequency (%) and median values [25th percentile (p25)‐75th percentile (p75)] were calculated for categorical and continuous variables, respectively. To control for imbalances in patient characteristics between cohorts, we calculated an exposure‐specific propensity score as the predicted probability of receiving the treatment of interest conditional upon the subjects’ baseline covariates using logistic regression models for the primary analysis and multinomial logistic regression models for the sensitivity analyses. We included the following covariates: baseline PASI (the score which was before and closest to the start of the treatment exposures within 6 months), smoking status (ever/never), current alcohol drinking (yes/no), alcohol consumption (units/week), obesity (≥30 kg/m^2^), age, gender, history of psoriatic arthritis (PsA), hypertension, diabetes, dyslipidemia, angina, previous treatment with ciclosporin, acitretin, fumaric acid esters and methotrexate. Covariate balance between the cohorts before and after propensity score overlap weighting was assessed using the expected percentage bias which is the difference in the outcome owing to the imbalance between each covariate taking into account the strength of the association between each covariate and the outcome. A maximum bias of 5% in either direction was considered an acceptable threshold. After generating propensity scores, overlap weights which were proportional to the probability of patients being assigned to the reference groups were calculated for only patients having predicted probabilities within the common support range. The common support range was defined as propensity scores of the treated groups overlapping the propensity scores of the reference groups.

Multiple imputation was used to address missing data on baseline PASI score, smoking status, current alcohol drinking, alcohol consumption and obesity using chained equations of 20 cycles to reduce bias. This method preserved the variability and uncertainty of missing data and avoids the loss of patients due to missing data and bias when compared with complete case analysis.^19^ The imputation model consisted of exposures, start year of exposure, log of censoring time for the outcome occurring during drug therapy; and during the extended window period, and whether patients experienced the outcomes during drug therapy; and during the extended window period, history of other heart diseases, concomitant drug therapies including ciclosporin, acitretin, fumaric acid esters and methotrexate; and the other covariates included in the propensity score model for the main analysis whilst the sensitivity analyses did not include concomitant methotrexate.

For each comparison (ustekinumab vs. TNFi for the primary analysis; and ustekinumab, etanercept or methotrexate vs. adalimumab for the secondary analyses) and for all outcomes, we calculated incidence rates (IRs), IR ratios, unadjusted, age and sex adjusted and overlap weighed hazard ratio (HRs) with 95% confidence intervals (CIs). We assessed the proportional hazards assumption by examining Schoenfeld residuals and confirming that it was not violated. All analyses were performed using Stata 14 (StataCorp LP, College Station, Texas, USA).

## Results

A total of 5468 patients were included in the main analysis [anti‐IL‐12/23 agent (ustekinumab): 951 and TNFi (adalimumab and etanercept): 4517; Fig. 1]. Patients in the ustekinumab group were more likely to be obese, but less likely to have either a history of PsA, currently drink alcohol or concomitantly receive methotrexate therapy, as shown in Table 1. The median (p25 and p75) follow‐up times for patients taking individual therapies were as follows: ustekinumab 1.76 (0.92–2.96) years and TNFi 1.69 (0.81–3.10) years for the analysis of events occurring during drug therapy; and ustekinumab 2.01 (1.16–3.21) years and TNFi 1.93 (1.05–3.34) years for the analysis of events occurring during the extended exposure window period.

**Figure 1:**
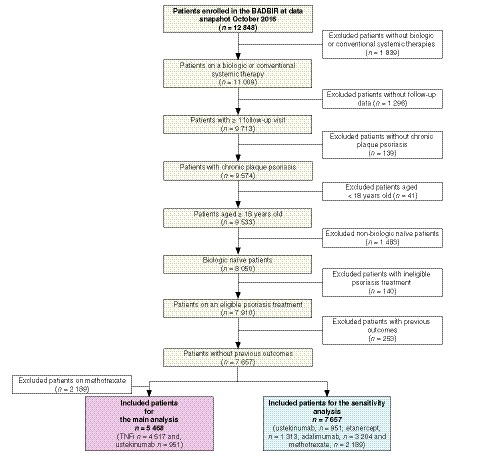
Patient selection.

**Table 1:** Baseline characteristics of patients receiving anti‐interleukin‐12/23 agent (ustekinumab) or TNFi (etanercept and adalimumab)

Characteristics	Ustekinumab	TNFi
Number of patients (*N *= 5468)	951	4517
Age (years; *N *= 5468)	45 (35–54) (*n *= 951)	44 (35.2–53) (*n *= 4517)
Sex, male (*N *= 5468)	590 (62.0) (*n *= 951)	2645 (58.6) (*n *= 4517)
Ethnicity, white (*N *= 5461)	853 (89.7) (*n *= 951)	4157 (92.2) (*n *= 4510)
BMI (kg/m^2^; *N *= 4983)	30.3 (26.2–35.7) (*n *= 851)	29.4 (25.9–33.8) (*n *= 4132)
Obese (BMI ≥30 kg/m^2^)	441 (51.8) (*n *= 851)	1922 (46.5) (*n *= 4132)
Ever smoke (yes/no; *N *= 4885)	599 (66.6) (*n *= 899)	2541 (63.8) (*n *= 3986)
Disease durations (years; *N *= 5417)	19 (11–30) (*n *= 943)	20 (12–29) (*n *= 4474)
PASI score (*N *= 4833)	14.6 (11.2–19.2) (*n *= 845)	14.1 (11.0–19.3) (*n *= 3988)
DLQI (*N *= 2949)	18 (12–24) (*n *= 460)	18 (13–24) (*n *= 2489)
**Comorbidities**		
No comorbidities	315 (33.1)	1356 (30.0)
Psoriatic arthritis	134 (14.1)	1035 (22.9)
Hypertension	241 (25.3)	1103 (24.4)
Diabetes mellitus	98 (10.3)	357 (7.9)
Dyslipidemia	98 (10.3)	435 (9.6)
Angina	20 (2.1)	57 (1.3)
Other heart diseases	23 (2.4)	80 (1.8)
Other comorbidities	512 (53.8)	2422 (53.6)
**Current alcohol drinking (** ***N*** ** = 4899)**	593 (65.7) (*n *= 903)	2854 (71.4) (*n *= 3996)
Alcohol units per week in patients consuming alcohol (*N *= 3382)	8 (3–15) (*n *= 584)	9 (3–16) (*n *= 2798)
Previous treatment of conventional systemic therapies		
Methotrexate	667 (70.1)	3124 (69.2)
Ciclosporin	540 (56.8)	2585 (57.2)
Acitretin	399 (42.0)	2008 (44.5)
Fumaric acid esters	165 (17.4)	879 (19.5)
**Concomitant therapies during drug therapy**		
Methotrexate	120 (12.6)	909 (20.1)
Ciclosporin	71 (7.5)	455 (10.1)
Acitretin	28 (2.9)	163 (3.6)
Fumaric acid esters	13 (1.4)	79 (1.8)
**Concomitant therapies during active use of the exposure or window period**		
Methotrexate	121 (12.7)	946 (20.9)
Ciclosporin	74 (7.8)	491 (10.9)
Acitretin	29 (3.1)	179 (4.0)
Fumaric acid esters	13 (1.4)	87 (1.9)

Data are *n* (%) or median (25th percentile‐75th percentile).

BMI, body mass index; DLQI, Dermatology Life Quality Index; PASI, Psoriasis Area Severity Index; TNFi, tumour necrosis factor‐α inhibitors.

Seven patients in the ustekinumab group experienced a major CVE during treatment with no additional patients experiencing such an outcome within 90 days after the last dose. For the TNFi cohort, 24 and 29 patients experienced major CVEs during drug therapy and during the extended exposure window period, respectively. The median times to onset of the major CVEs in both groups were about 1 year during either drug therapy or the extended exposure window period (Table 2).

**Table 2:** Incidence rates and incidence rate ratios among patients receiving anti‐interleukin‐12/23 agent (ustekinumab) or TNFi (etanercept and adalimumab)

	Ustekinumab	TNFi
**Outcome during drug therapy**		
Total patient‐years	1936.56	9757.22
Patient‐years of follow‐up (median, p25–p75)	1.76 (0.92–2.96)	1.69 (0.81–3.10)
Number of major cardiovascular events	7	24
Incidence rate per 1000 patient‐years (95% CI)	3.61 (1.72–7.58)	2.46 (1.65–3.67)
Incidence rate ratio	1.47 (0.53–3.52)	Reference
Duration between the start of exposure to development of the outcome (years; median, p25–p75; only patients experiencing the outcome)	1.06 (0.59–1.94)	1.19 (0.50–2.14)
**Outcome during drug therapy plus grace period (90 days)**		
Total patient‐years	2167.61	10 858.90
Patient‐years of follow‐up (median, p25–p75)	2.01 (1.16–3.21)	1.93 (1.05–3.34)
Number of major cardiovascular events	7	29
Incidence rate per 1000 patient‐years (95% CI)	3.23 (1.54–6.77)	2.67 (1.86–3.84)
Incidence rate ratio	1.21 (0.45–2.82)	Reference
Duration between the start of exposure to development of the outcome (years; median, p25–p75; only patients experiencing the outcome)	1.06 (0.59–1.94)	1.06 (0.47–1.98)

95% CI, 95% confidence interval; p25–p75, 25th percentile‐75th percentile; TNFi, tumour necrosis factor‐α inhibitors.

### Incidence rates of major cardiovascular events

The IRs of major CVEs associated with ustekinumab therapy for both periods were numerically but not statistically significantly higher than those associated with TNFi. Crude IRs (95% CI) in the ustekinumab and TNFi groups were 3.61 (1.72–7.58) and 2.46 (1.65–3.67) per 1000 patient‐years, respectively, for the outcome during drug therapy; and 3.23 (1.54–6.77) and 2.67 (1.86–3.84) per 1000 patient‐years, respectively, for the extended exposure window period (Table 2).

### Comparative risks of major cardiovascular events

**Figure 2:**
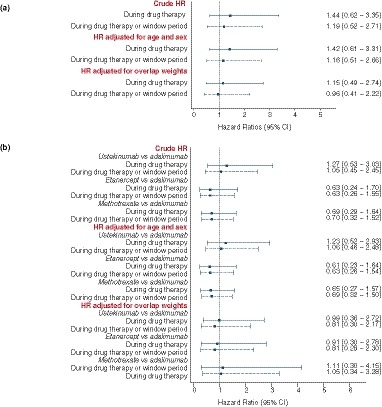
Crude and adjusted hazard ratios (95% confidence interval) for major cardiovascular events associated with different psoriasis therapies. (a) Comparison of anti‐interleukin‐12/23 agent (ustekinumab) with tumour necrosis factor‐α inhibitors (referent group). (b) Comparisons of ustekinumab, etanercept or methotrexate with adalimumab (referent group).

**Figure 3:**
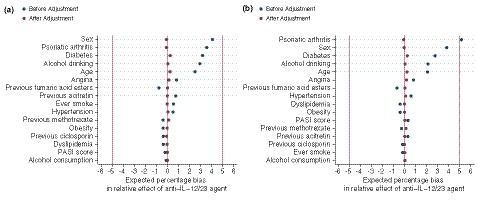
Distribution of confounders between anti‐interleukin‐12/23 agent (ustekinumab) and tumour necrosis factor‐α inhibitors (referent) patients before creating propensity score and after overlap weighting by propensity score. (a) Outcomes occurring during drug therapy. (b) Outcomes occurring during drug therapy plus grace period (90 days).

The unadjusted and age‐sex adjusted HRs showed no difference in the risk of major CVEs between patients treated with ustekinumab and TNFi therapies. In the propensity score‐adjusted analysis, there was similarly no difference in the risk of major CVEs occurring during both periods (Fig. 2a). The baseline characteristics of the treatment cohorts were comparable after applying the overlap weights using the propensity score method as shown in Fig. 3.

### Secondary analyses comparing the risk of major CVEs associated with individual therapies

A total of 7657 patients were included in the secondary analyses (ustekinumab, 951; etanercept, 1313; methotrexate, 2189 and: adalimumab, 3204). The proportions of patients with PsA in the ustekinumab (14.1%) and methotrexate (8.9%) groups were lower than in the adalimumab (23.3%) or etanercept (21.9%) groups, as shown in Table S2 (Supporting Information). The ustekinumab, etanercept and adalimumab cohorts had longer durations of follow‐up than the methotrexate group (Table 3).

**Table 3:** Incidence rates and incidence rate ratios among patients receiving ustekinumab, etanercept, methotrexate or adalimumab

	Ustekinumab	Etanercept	Methotrexate	Adalimumab
**Outcome during drug therapy**				
Total patient‐years	1936.56	2905.99	3650.81	6851.23
Patient‐years of follow‐up (median, p25–p75)	1.76 (0.92–2.96)	1.67 (0.69–3.20)	1.18 (0.59–2.29)	1.69 (0.84–3.07)
Number of major cardiovascular events	7	5	7	19
Incidence rate per 1000 patient‐years (95% CI)	3.61 (1.72–7.58)	1.72 (0.72–4.13)	1.92 (0.91–4.02)	2.77 (1.77–4.35)
Incidence rate ratio	1.30 (0.46–3.24)	0.62 (0.18–1.72)	0.69 (0.25–1.72)	Reference
Incidence rate ratio	1.89 (0.56–6.30)	0.90 (0.22–3.28)	Reference	1.45 (0.58–4.07)
Duration between the start of exposure to development of the outcome (years; median, p25–p75; only patients experiencing the outcome)	1.06 (0.59–1.94)	1.29 (1.08–1.82)	0.99 (0.86–1.60)	0.90 (0.46–2.29)
**Outcome during drug therapy plus grace period (90 days)**				
Total patient‐years	2167.61	3226.03	4185.94	7632.87
Patient‐years of follow‐up (median, p25–p75)	2.01 (1.16–3.21)	1.92 (0.93–3.45)	1.43 (0.84–2.53)	1.94 (1.09–3.32)
Number of major cardiovascular events	7	6	9	23
Incidence rate per 1000 patient‐years (95% CI)	3.23 (1.54–6.77)	1.86 (0.84–4.14)	2.15 (1.12–4.13)	3.01 (2.00–4.53)
Incidence rate ratio	1.07 (0.39–2.58)	0.62 (0.21–1.56)	0.71 (0.29–1.60)	Reference
Incidence rate ratio	1.50 (0.48–4.53)	0.87 (0.25–2.72)	Reference	1.40 (0.62–3.44)
Duration between the start of exposure to development of the outcome (years; median, p25–p75; only patients experiencing the outcome)	1.06 (0.59–1.94)	1.19 (1.06–1.82)	0.99 (0.86–1.60)	0.90 (0.44–2.29)

95% CI, 95% confidence interval; p25–p75, 25th percentile‐75th percentile.

During drug therapy, major CVEs occurred in 7, 5, 7 and 19 patients receiving ustekinumab, etanercept, methotrexate and adalimumab, respectively; during the extended exposure window period, major CVEs occurred in 7, 6, 9 and 23 patients, respectively. The IRs associated with exposure to ustekinumab were numerically higher than those associated with adalimumab and methotrexate but these differences were not significant. The median times to onset of major CVEs in all groups and analyses were about 1 year but etanercept had the longest onset of major CVEs compared with the other groups (Table 3).

The proportionality test for all comparisons and both analysis times showed no violation of the proportional hazard assumptions. Moreover, the expected percentage bias achieved a good balance in all analyses, after adjusted for overlap weights by propensity score (Figs S1–S3, Supporting Information).

There were no significant differences in the risk for major CVE occurring during drug therapy or the extended exposure window period when patients using ustekinumab, etanercept or methotrexate were compared with those using adalimumab as shown in (Fig. 2b).

## Discussion

In this large prospective cohort study, we found no significant differences in the risk of major CVEs between biologic therapies in adult patients with chronic plaque psoriasis. Moreover, the risk of major CVEs for methotrexate was not significantly different from adalimumab. These findings are derived from propensity score‐adjusted models taking into account a range of important CV risk factors. Our findings were consistent for separate analyses comparing the risk of major CVEs both during therapy and for an extended exposure window period.

Earlier observational studies had a number of differences which make comparison with our study difficult: notably, different comparators and definitions of CV outcomes, including participants with prior CVEs in the studies, and not controlling for some important CV risk factors^11^, ^12^, ^13^, ^14^, ^15^ (Table S3, Supporting Information). The results of these previous studies suggested benefits of biologic therapies in relation to risk of CV outcomes. One study suggested that TNFi‐treated patients (adalimumab, etanercept and infliximab; *n *= 9148) had a significantly lower risk of composite and individual CVEs (MI; stroke or transient ischaemic attack; or unstable angina) when compared with those treated with methotrexate (*n *= 8581).^11^ In addition, two cohort studies suggested that TNFi (*n*1 = 1463 and *n*2 = 11 410) significantly decreased the risk of major adverse CVEs when compared with topical therapies (*n *= 13 112) and the risk of major CVEs (MI; stroke or transient ischaemic attack; or unstable angina; which is different definition than we used in this current study) when compared with phototherapy (*n *= 12 433).^14^, ^15^ Another study defined CVEs as composite MI, stroke and CV death. It found a significantly lower risk of CVEs in TNFi (*n *= 959) and methotrexate (*n *= 3564)‐treated groups, whilst the risk in those treated with ustekinumab (*n *= 178) was similar to those using other therapies (topical, phototherapy and climate therapy; *n *= 3961).^13^ Since the sample size of the ustekinumab group was very small in this earlier study, it is unlikely that any difference in the risk of CVEs would be detected for this comparison. In line with our findings, an earlier cohort study found that patients treated with biologic therapies (including ustekinumab, adalimumab, etanercept, alefacept and efalizumab; *n *= 7682 at enrolment) had a similar risk of CVEs (non‐fatal‐MI, non‐fatal‐stroke and CV death) when compared to those treated with non‐biologic agents (*n *= 5576 at enrolment).^12^ A recent large cohort study compared ustekinumab (*n *= 9071) with TNFi (adalimumab, etanercept, infliximab, certolizumab or golimumab; *n *= 50 957) among patients with psoriasis or PsA to examine the risk of atrial fibrillation or major adverse CVEs.^20^ In line with our findings, this study found no significant difference in the risk of CVEs outcomes. Of related interest, two RCTs examining the impact of adalimumab (TNFi) on aortic vascular inflammation in patients with moderate–severe psoriasis also reported that adalimumab did not improve aortic vascular inflammation after 52 weeks of treatment.^21^, ^22^

Power calculation is used to inform how well we could characterize nature in the future given in a certain situation and statistical study design.^23^ Since this study explored the relationship between major CVEs and biologic therapies for the treatment of psoriasis using the real data and there was no significant difference in the risk of major CVEs for all comparisons, it is pointless to calculate post hoc power calculation. It does not yield additional insights.^23^

Our study has several important strengths. Firstly, we reduced potential bias by using a new‐user study design for the biologic cohorts^24^ and propensity score techniques for examining the impact of biologic therapies on risk of major CVEs. The propensity score technique adequately controlled for measured CV confounders between comparison groups. Secondly, we excluded patients who had experienced prior major CVEs to further minimize bias.

We also acknowledge some study limitations. First, although we controlled for measured confounders including the most important CV risk factors, we cannot exclude the effects of residual confounding due to other unmeasured variables such as physical activity and dietary factors. The propensity score technique cannot address this limitation. Second, some aspects of CV risk factor management may be specific to this national cohort, and therefore, the results may not be generalizable to patients managed in different healthcare systems. Third, the small numbers of major CVEs and participants and the limited follow‐up may have had an impact on the power for these analyses as seen in HRs with 95% CIs. Moreover, the impact of biologic therapies on the risk of major CVEs may change over time. Our findings may serve as hypothesis generating for future studies. Therefore, continued surveillance of the risk of major CVEs in more patients with plaque psoriasis with longer follow‐up is needed.

## Conclusion

Overall, we found no difference in the risk of major CVEs between etanercept, adalimumab and ustekinumab in adult patients with moderate–severe plaque psoriasis following short‐to‐medium‐term exposure. The impact of biologic therapies or methotrexate on the risk of major CVEs in patients with psoriasis may take longer to manifest. Thus, future comparative studies with longer follow‐up and additional data on CV risk factors will be helpful for continued surveillance of major CVEs in patients with psoriasis exposed to biologic therapies.

## Supporting information


**Table S1.** Potential adverse event terms
**Table S2.** Baseline characteristics of patients receiving ustekinumab, etanercept, methotrexate or adalimumab.
**Table S3.** Cohort studies examining the association between biologic therapies and cardiovascular events.
**Figure S1.** Distribution of confounders between ustekinumab and adalimumab (referent) patients before creating propensity score and after overlap weighting by propensity score.
**Figure S2.** Distribution of confounders between etanercept and adalimumab (referent) patients before creating propensity score and after overlap weighting by propensity score.
**Figure S3.** Distribution of confounders between methotrexate and adalimumab (referent) patients before creating propensity score and after overlap weighting by propensity score.
