# Supplementary material for: Risk of major cardiovascular events in patients with psoriasis receiving biologic therapies: a prospective cohort study
Source: J Eur Acad Dermatol Venereol. 2019 Nov 19;34(4):769–78. doi: 10.1111/jdv.16018 (PMC7155017; doi:10.1111/jdv.16018)
Supplement: Supplementary file 1 — Table S1. Potential adverse event terms Table S2. Baseline characteristics of patients receiving ustekinumab, etanercept, methotrexate or adalimumab. Table S3. Cohort studies examining the association between biologic therapies and cardiovascular events. Figure S1. Distribution of confounders between ustekinumab and adalimumab (referent) patients before creating propensity score and after overlap weighting by propensity score. Figure S2. Distribution of confounders between etanercept and adalimumab (referent) patients before creating propensity score and after overlap weighting by propensity score. Figure S3. Distribution of confounders between methotrexate and adalimumab (referent) patients before creating propensity score and after overlap weighting by propensity score. [file JDV-34-769-s001.docx]

**Supplementary**

**Table S1. Potential adverse event terms**

| **System organ class (MedDRA system organ class)** | **Adverse event preferred term (MedDRA preferred term)** |
| --- | --- |
| Cardiac disorders | Acute coronary syndrome  Acute myocardial infarction  Angina unstable  Myocardial infarction |
| General disorders and administration site conditions | Death |
| Nervous system disorders | Carotid artery occlusion  Cerebellar haemorrhage  Cerebral haemorrhage  Cerebral infarction  Cerebrovascular accident  Intracranial haemorrhage  Hemiparesis  Hemiplegia  Ischaemic stroke  Lacunar infarction  Monoplegia  Subarachnoid haemorrhage  Subdural haematoma  Thalamic infarction |

**Table S2. Baseline characteristics of patients receiving ustekinumab, etanercept, methotrexate or adalimumab**

| **Characteristics** | **Ustekinumab** | **Etanercept** | **Methotrexate** | **Adalimumab** |
| --- | --- | --- | --- | --- |
| Number of patients (*N*=7,657) | 951 | 1,313 | 2,189 | 3,204 |
| Age (years) (*N*=7,657) | 45 (35 - 54)  (*n*=951) | 44.95 (36 - 53) (*n*=1,313) | 43 (33 - 53)  (*n* = 2,189) | 44 (35 - 53)  (*n*=3,204) |
| Sex, male (*N*=7,657) | 590 (62.0) | 758 (57.7) | 1,127 (51.5) | 1,887 (58.9) |
| Ethnicity, white (*N*=7,647) | 853 (89.7)  (*n*=951) | 1,209 (92.3)  (*n*=1,310) | 1,970 (90.1)  (*n*=2,186) | 2,948 (92.1)  (*n*=3,200) |
| BMI (kg/m^2^) (*N*=6,964) | 30.3  (26.2 – 35.7)  (*n*=851) | 29.2  (25.8 –34.1)  (*n*=1,193) | 28.7  (25.2 – 33.2)  (*n*=1,981) | 29.4  (26.0 – 33.7)  (*n*=2,939) |
| Obese (BMI≥30kg/m^2^)  (*N*=6,964) | 441 (51.8)  (*n*=851) | 546 (45.8)  (*n*=1,193) | 824 (41.6)  (*n*=1,981) | 1,376 (46.8)  (*n*=2,939) |
| Ever smoke (yes/no) (*N*=6,873) | 599 (66.6)  (*n*=899) | 656 (61.8)  (*n*=1,061) | 1,345 (67.7)  (*n*=1,988) | 1,885 (64.4)  (*n*=2,925) |
| Disease duration (years) (*N*=7,593) | 19 (11 - 30 )  (*n*=943) | 20 (12 - 30)  (*n*=1,307) | 17 (8 - 27)  (*n*=2,176) | 20 (12 - 29)  (*n*=3,167) |
| PASI score (*N*= 6,384) | 14.6  (11.2 – 19.2) (*n*=845) | 13.8  (10.8 – 18.6)  (*n*=1,103) | 13  (10.3 – 17.8)  (*n*=1,551) | 14.2  (11 – 19.5)  (*n*=2,885) |
| DLQI (*N*=4,516) | 18 (12 - 24)  (*n*=460) | 18 (12 - 23)  (*n*=719 | 15 (11 - 21)  (*n*=1,567) | 19 (13 - 24)  (*n*= 1,770) |
| Comorbidities (*N*=7,657) |  |  |  |  |
| No comorbidities | **315** (3**3**.1) | 367 (28.0) | 844 (38.6) | 989 (30.9) |
| Psoriatic arthritis | 134 (14.1) | 288 (21.9) | 194 (8.9) | 747 (23.3) |
| Hypertension | 241 (25.3) | 360 (27.4) | 380 (17.4) | 743 (23.2) |
| Diabetes mellitus | 98 (10.3) | 114 (8.7) | 140 (6.4) | 243 (7.6) |
| Dyslipidemia | 98 (10.3) | 126 (9.6) | 170 (7.8) | 309 (9.6) |
| Angina | 20 (2.1) | 16 (1.2) | 30 (1.4) | 41 (1.3) |
| Other heart diseases | 23 (2.4) | 17 (1.3) | 51 (2.3) | 63 (2.0) |
| Other comorbidities | 512 (53.8) | 692 (52.7) | 1,101 (50.3) | 1,730 (54.0) |
| Current alcohol drinking (*N*=6,881) | 593 (65.7)  (*n*=903) | 749 (70.5)  (*n*=1,062) | 1,272 (64.2)  (*n*=1,982) | 2,105 (71.8)  (*n*=2,934) |
| Alcohol units per week in patients consuming alcohol (*N*=4,628) | 8 (3 - 15)  (*n*=584) | 10 (4 - 18)  (*n*=720) | 6 (2 - 12)  (*n*=1,246) | 8 (3 - 15)  (*n*=2,078) |
| Previous treatment of conventional systemic therapies | | | |  |
| Methotrexate | 667 (70.1) | 865 (65.9) | 250 (11.4) | 2,259 (70.5) |
| Ciclosporin | 540 (56.8) | 684 (52.1) | 497 (22.7) | 1,901 (59.3) |
| Acitretin | 399 (42.0) | 593 (45.2) | 610 (27.9) | 1,415 (44.2) |
| Fumaric acid esters | 165 (17.4) | 331 (25.2) | 132 (6.0) | 548 (17.1) |
| Concomitant therapies during drug therapy | | | |  |
| Ciclosporin | 71 (7.5) | 125 (9.5) | 266 (12.2) | 330 (10.3) |
| Acitretin | 28 (2.9) | 59 (4.5) | 91 (4.2) | 104 (3.3) |
| Fumaric acid esters | 13 (1.4) | 33 (2.5) | 31 (1.4) | 46 (1.5) |
| Concomitant therapies during active use of the exposure or window period | | | | |
| Ciclosporin | 74 (7.8) | 132 (10.1) | 409 (18.7) | 359 (11.2) |
| Acitretin | 29 (3.0) | 66 (5.0) | 185 (8.5) | 113 (3.5) |
| Fumaric acid esters | 13 (1.4) | 36 (2.7) | 93 (4.3) | 51 (1.6) |

**Data are n (%) or median (25^th^ percentile - 75^th^ percentile)**

**Abbreviations:** BMI, body mass index; PASI, Psoriasis Area Severity Index; DLQI, Dermatology Life Quality Index

**Table S3 Cohort studies examining the association between biologic therapies and cardiovascular events**

| **Authors, year** | **Study population and comparison groups** | **Outcome** | **Study design** | **Results** | **Advantages** | **Disadvantages** |
| --- | --- | --- | --- | --- | --- | --- |
| Wu et.al., 2017^1^ | Adult psoriasis patients aged ≥ 18 years old  TNFi (n=9,148) vs. methotrexate (n=8,581) (reference group) | Major CVEs (MI; stroke or transient ischemic attack; or unstable angina) | Retrospective cohort study  Using health claims data source  Cox proportional hazards models were used and controlled confounders age, sex, region, health plan, Charlson-Quan comorbidity index, prior major CVEs, comorbidities frequently related to psoriasis, prior use of psoriasis systemic therapies, non-psoriasis medicines related to an increased risk of major CVEs and the number of medical visits. | **Major CVEs**  Adjusted HR (95%CI) = 0.55 (0.45 – 0.67) | - It involved a large number of participants receiving TNFi..  - It controlled a number of confounders. | - The results of the study might be biased due to inappropriate study design which could introduce bias such as using an inappropriate reference group (methotrexate) which was used in patients with lower severity of psoriasis compared with patients receiving TNFi (The more severity of psoriasis is associated with an increased risk of CVEs), not excluding patients with the previous CVEs (these patients were more likely to develop the recurrent CVEs), not using a new-user study design (to minimise the influence of the previous psoriasis biologic therapies on the risk of CVEs) and not controlling some important CVconfounders (e.g. severity of psoriasis, smoking and alcohol drinking)  - It was not clear what TNFi were included in this study. Different TNFi might have an effect on the risk of CV outcomes differently.  - When patients received > 1 TNFi, the index date was randomly selected from all TNFi initiation dates. For patients receiving methotrexate, the index date was randomly selected from all dates of a prescription fill for methotrexate.  Thus, the exposure period might not represent the real exposure time of the exposures. |
| Gottlieb et.al., 2014^2^ | Patients with psoriasis  Infliximab (n=6,028), ustekinumab (n=7,047) or other biologic therapies (adalimumab, etanercept, alefacept and efalizumab) (n=13,167) vs non-biologic therapies (reference group) | Major adverse CVEs (non-fatal cerebrovascular accident, non-fatal MI and CV death) | Cohort study using a large intercontinental, psoriasis registry  Cox proportional hazards models were used and controlled confounders history of CV disease; age; gender; ethnicity; body mass index; duration of disease; PsA; history of biologic therapies and immunomodulators before the enrolment; infliximab, other biologic therapies (adalimumab, etanercept, alefacept and efalizumab) and immunomodulators after the enrolment. | **Major adverse CVEs**  **Adjusted HR (95%CI)**  **For Inflixima**b = 0.965 (0.439 – 2.121)  **For all biologic therapies except infliximab** = 1.127 (0.713 – 1.780),  **For infliximab and other biologic therapies** = 0.983 (0.329 – 2.931) | - - It was a large intercontinental cohort study involving a number of patients with psoriasis. - It used a psoriasis registry which was designed to collect safety information and clinical status among psoriasis patients receiving systemic therapies for the treatment of psoriasis^3^ - - It collected data from real world practice using the registry for their analyses. Thus, it reflected real-life practice from the use of biologic therapies. | - The study design of this study was not good enough to control confounders such as not using new user study design, not controlling some important CV risk factors (e.g. diabetes and smoking), including psoriasis patients with prior CV events. These patients were more likely to develop recurrent CVEs compared with patients without prior CVEs. These could lead to biased results. - To analyse major adverse CVEs, it used an ever-exposed method. The results of the study relating to major adverse CVEs might be biased if patients discontinued for a long period since other factors could contribute to the development of the CV outcomes. - This study analysed data from currently non-licenced biologic therapies which are alefacept and efalizumab. - CV death which was defined as a major adverse CVE was justified by physician review and other data. It did not clear what CV events were included. - Some researchers were employees of a pharmaceutical company producing a biologic therapy. They could have an influence on designing the study and analysing the data which might lead to biased results. |
| Ahlehoff et.al., 2015^4^ | Patients with severe psoriasis aged ≥ 18 years  Biologic therapies (n=1,137), TNFi (n=959), anti-IL-12/23 agent (ustekinumab) (n=178)  vs other therapies (topical therapy and climate therapy) (n=3,961) (reference group) | Composited CVEs (MI, stroke and CV death) | Registry-based cohort study  Cox regression models controlled confounders age, sex, year of inclusion, baseline use of medicines, and comorbidities. | **Composited CVEs**  **Adjusted HR (95%CI)**  **For biologic therapies** = 0.58 (0.30 – 1.10)  **For TNFi** = 0.46 (0.22 – 0.98)  **For anti-IL-12/23 agent (ustekinumab)** = 1.52 (0.47 – 4.94) | -This study used real-world practice data for analyses and took into account some CV confounders when analyses. | -The numbers of patients receiving biologic therapies, TNFi and ustekinumab were small.  -This study did not clearly mention what biologic therapies were included in the biologic therapies cohort.  - The results of the study might be biased due to inappropriate study design which could introduce bias such as using an inappropriate reference group (topical therapy and climate therapy) which was used in patients with lower severity of psoriasis (The more severity of psoriasis is associated with an increased risk of CVEs), not excluding patients with prior composited CVEs who have higher likelihood of recurrent CVEs, not using a new-user study design (to minimise the influence of the previous psoriasis biologic therapies on the risk of CVEs) and not controlling some important CVconfounders (e.g. severity of psoriasis and PsA). |
| Wu et.al., 2018^5^ | Patients with psoriasis or PsA aged ≥ 18 years  Exclusion criterion: history of prior major adverse CVEs  TNFi (adalimumab, etanercept or infliximab) (n=1,463) or oral agents(acitretin, apremilast, cyclosporine or methotrexate) /phototherapy (n=3,579) vs topical therapy (n=13,112) (reference group) | Major adverse CVEs (MI, stroke and CV death) | Retrospective cohort study using the KPSC health plan  Propensity score technique was used to control confounders age, sex, race/ethnicity, history of smoking or alcohol drinking, use of clopidogrel,anti-hypertensive drugs, anti-hyperlipidaemia drugs or anti-coagulants. | **Adjusted HR (95%CI) when compared with topical therapy** **For TNFi** = 0.80 (0.66 – 0.98)  **For oral/phototherapy** = 1.19 (0.99 – 1.42) | -It controlled a number of confounders using propensity score technique.  -It excluded psoriasis patients with prior major adverse CVEs who had higher likelihood of recurrent the CVE. | - The results of the study might be biased due to inappropriate study design which could introduce bias such as using an inappropriate reference group (topical therapy) which was used in patients with lower severity of psoriasis (The more severity of psoriasis is associated with an increased risk of CVEs), not controlling some important CV confounders (e.g. severity of psoriasis, PsA)  -The number of patients treated with TNFi was small and individual TNFi were not separately analysed. Different TNFi might have an effect on the development of the major CVEs differently. |
| Wu et.al., 2018^6^ | Adult patients with psoriasis aged ≥ 18 years  TNFi (adalimumab, etanercept or infliximab) (n=11,410) vs phototherapy (n=12,433) (reference group) | Major CVEs (MI, stroke, transient ischemic attack or unstable angina) | Cohort using a US administrative claims database  Cox proportional hazards models were used and controlled confounders age, sex, Charlson comorbitidy index, previous use of psoriasis therapies and other therapies relating to an increased risk of CVEs and psoriasis associated with comorbidities with statistically significant differences between comparison groups at baseline. | **Major CVEs**  Adjusted HR (95%CI) for TNFi = 0.77 (0.60 – 0.99) | -It involved a large number of participants.  - It controlled a number of confounders. | -The results of this study might be biased due to inappropriate study design which could introduce bias such as using an inappropriate reference group (phototherapy) which was recommended for patients with lower severity of psoriasis compared with biologic therapies (The more severity of psoriasis is associated with an increased risk of CVEs), not controlling some important confounders (e.g. severity of psoriasis, smoking and alcohol drinking)  - A pharmaceutical company marketing a biologic therapy for the treatment of psoriasis involved in the study design of this study and interpretation of the data. This could lead to biased results. |
| Lee et.al., 2019^7^ | Patients with psoriasis or PsA aged ≥ 18 years  Exclusion criteria: history AF or receiving anti-arrhythmic or anti-coagulant therapy  Ustekinumab (n=9,071) vs TNFi (50,957 (adalimumab, etanercept, infliximab, certolizumab or golimumab) (reference group) | AF or major adverse CVEs (MI, stroke or coronary revascularization) | Cohort  Propensity score fine stratification and weighting was used to control potential confounding and patients having propensity score in overlapping area were included in this study. It controlled confounders age, sex, number of clinical visits for psoriasis and PsA, psoriasis therapies, comorbidities (e.g. diabetes hyperlipidaemia, stroke), other therapies (e.g.insulin, anti-hypertensive drugs), health care utilization measures, obesity, smoking and alcohol drinking  Two US Commercial insurance databases were used for this study. | **For AF**  **Adjusted HR (95%CI) =** 1.08 (0.76 – 1.54)  **For major adverse CVEs**  = 1.10 (0.80 – 1.52) | - - It used two large U.S. commercial insurance databases involving a large number of patients with psoriasis and PsA.   - It used a good study design such as using an active comparator (TNFi), a new-user study design, propensity score technique fine stratification to control a number of confounders. | - This study did not exclude patients with prior major adverse CVEs. These patients were more likely to experience recurrent the CVEs. - Due to unavailable data, this study did not control some confounders which are related to the development of CV outcomes such as the severity of psoriasis (e.g. PASI score), the proportionality of patients with PsA. - Follow-up time of patients treated with TNFi could be continuously counted when patients treated with different TNFis. Thus, this study could not distinguish the impact of different TNFi on the development of major adverse CVEs. - This study did not use propensity score technique to control only covariates which were strongly related to the outcome; or the outcome and the exposure such as asthma. Thus, these could not reduce the variance of estimated exposure effects.^8^ |

**Abbreviations:** AF = atrial fibrillation, CI = confidence interval, CV = cardiovascular, CVEs = cardiovascular events, HR = hazard ratio, MI = myocardial infarction, PsA = psoriatic arthritis, TNFi = tumour necrosis factor-α inhibitors

**Figure S1. Distribution of confounders between ustekinumab and adalimumab (referent) patients before creating propensity score and after overlap weighting by propensity score**

1. **Outcomes occurring during drug therapy**

**Abbreviation:** PASI, Psoriasis area and severity index

1. **Outcomes occurring during drug therapy plus grace period (90 days)**

**Abbreviation:** PASI, Psoriasis area and severity index

**Figure S2. Distribution of confounders between etanercept and adalimumab (referent) patients before creating propensity score and after overlap weighting by propensity score**

1. **Outcomes occurring during drug therapy**

**Abbreviation:** PASI, Psoriasis area and severity index

**(b) Outcomes occurring during drug therapy plus grace period (90 days)**

**Abbreviation:** PASI, Psoriasis area and severity index

**Figure S3. Distribution of confounders between methotrexate and adalimumab (referent) patients before creating propensity score and after overlap weighting by propensity score**

1. **Outcomes occurring during drug therapy**

**Abbreviation:** PASI, Psoriasis area and severity index

1. **Outcomes occurring during drug therapy plus grace period (90 days)**

**Abbreviation:** PASI, Psoriasis area and severity index

**References**

1. Wu JJ, Guérin A, Sundaram M, Dea K, Cloutier M, Mulani P. Cardiovascular event risk assessment in psoriasis patients treated with tumor necrosis factor-α inhibitors versus methotrexate. *J Am Acad Dermatol*. 2017;**76**:81-90.

2. Gottlieb AB, Kalb RE, Langley RG, et al. Safety observations in 12095 patients with psoriasis enrolled in an international registry (PSOLAR): experience with infliximab and other systemic and biologic therapies. *J Drugs Dermatology*. 2014;**13**:1441-1448.

3. Papp K, Strober B, Augustin M, et al. PSOLAR: design, utility, and preliminary results of a prospective, international, disease-based registry of patients with psoriasis who are receiving, or are candidates for, conventional systemic treatments or biologic agents. *J Drugs Dermatol*. 2012;**11**:1210-1217.

4. Ahlehoff O, Skov L, Gislason G, et al. Cardiovascular outcomes and systemic anti-inflammatory drugs in patients with severe psoriasis: 5-year follow-up of a Danish nationwide cohort. *J Eur Acad Dermatology Venereol*. 2015;**29**:1128-1134.

5. Wu JJ, Joshi AA, Reddy SP, et al. Anti-inflammatory therapy with tumor necrosis factor inhibitors is associated with reduced risk of major adverse cardiovascular events in psoriasis. *J Eur Acad Dermatology Venereol*. 2018;**32**:1320-1326.

6. Wu JJ, Sundaram M, Cloutier M, et al. The risk of cardiovascular events in psoriasis patients treated with tumor necrosis factor–α inhibitors versus phototherapy: an observational cohort study. *J Am Acad Dermatol*. 2018;**79**:60-68.

7. Lee MP, Desai RJ, Jin Y, Brill G, Ogdie A, Kim SC. Association of ustekinumab vs TNF inhibitor therapy with risk of atrial fibrillation and cardiovascular events in patients with psoriasis or psoriatic arthritis. *JAMA Dermatology*. 2019;**155**:700-707.

8. Brookhart MA, Schneeweiss S, Rothman KJ, Glynn RJ, Avorn J, Stürmer T. Variable selection for propensity score models. *Am J Epidemiol*. 2006;**163**:1149-1156.
